# A New Method to Extract Health-Related Quality of Life Data From Social Media Testimonies: Algorithm Development and Validation

**DOI:** 10.2196/31528

**Published:** 2022-01-28

**Authors:** Simon Renner, Tom Marty, Mickaïl Khadhar, Pierre Foulquié, Paméla Voillot, Adel Mebarki, Ilaria Montagni, Nathalie Texier, Stéphane Schück

**Affiliations:** 1 Kap Code Paris France; 2 Bordeaux Population Health Research Center, UMR 1219 Bordeaux University Inserm Bordeaux France

**Keywords:** health-related quality of life, social media use, measures, real world, natural language processing, social media, NLP, infoveillance, quality of life, digital health, social listening

## Abstract

**Background:**

Monitoring social media has been shown to be a useful means to capture patients’ opinions and feelings about medical issues, ranging from diseases to treatments. Health-related quality of life (HRQoL) is a useful indicator of overall patients’ health, which can be captured online.

**Objective:**

This study aimed to describe a social media listening algorithm able to detect the impact of diseases or treatments on specific dimensions of HRQoL based on posts written by patients in social media and forums.

**Methods:**

Using a web crawler, 19 forums in France were harvested, and messages related to patients’ experience with disease or treatment were specifically collected. The SF-36 (Short Form Health Survey) and EQ-5D (Euro Quality of Life 5 Dimensions) HRQoL surveys were mixed and adapted for a tailored social media listening system. This was carried out to better capture the variety of expression on social media, resulting in 5 dimensions of the HRQoL, which are physical, psychological, activity-based, social, and financial. Models were trained using cross-validation and hyperparameter optimization. Oversampling was used to increase the infrequent dimension: after annotation, SMOTE (synthetic minority oversampling technique) was used to balance the proportions of the dimensions among messages.

**Results:**

The training set was composed of 1399 messages, randomly taken from a batch of 20,000 health-related messages coming from forums. The algorithm was able to detect a general impact on HRQoL (sensitivity of 0.83 and specificity of 0.74), a physical impact (0.67 and 0.76), a psychic impact (0.82 and 0.60), an activity-related impact (0.73 and 0.78), a relational impact (0.73 and 0.70), and a financial impact (0.79 and 0.74).

**Conclusions:**

The development of an innovative method to extract health data from social media as real time assessment of patients’ HRQoL is useful to a patient-centered medical care. As a source of real-world data, social media provide a complementary point of view to understand patients’ concerns and unmet needs, as well as shedding light on how diseases and treatments can be a burden in their daily lives.

## Introduction

Most people use the internet regularly to research and discuss health-related topics. Patients give and receive advice on their diseases and treatments in online forums and social media platforms [1]. These messages are massive, continuously generated, and easy to access [2]. This type of information is direct, genuine, and authentic, offering access to new real-world data, which can facilitate the understanding of patients’ perspectives. As the internet offers anonymity, patients talk about their fears and concerns and share details about their diseases and treatments, which can inform health public authorities, pharmaceutical companies, and other health professionals and institutions [3]. Thus, social media are a large and diverse source of information nurtured by continuous exchanges and interactions, ranging from commenting on posts to sharing of opinions.

The World Health Organization defines quality of life (QoL) as individuals’ perception of their place in life in the context of the culture and the value system in which they live, as well as in relation to their objectives, expectations, standards, and concerns. This is a broad conceptual field, encompassing, in a complex way, a person’s physical health, psychological state, level of independence, social relationships, personal beliefs, and relationship with the specificities of the surrounding environment [4]. When the study of QoL is restricted to health-related effects, one can refer to them as health-related quality of life (HRQoL) [5]. Therefore, HRQoL is a multidimensional concept focusing on the impact health and diseases have on QoL [6,7]. This concept is mainly used in epidemiology and cost-effectiveness analysis [8].

Several instruments have been developed to quantitatively measure individuals’ HRQoL [9]. Among them, the EQ-5D (Euro Quality of Life 5 Dimensions) and SF-36 (Short Form Health Survey) have been used in medical practice for more than 20 years [10,11]. They are designed to be self-completed by patients. Nonetheless, these surveys are not adapted to the amount of qualitative information on QoL contained within the free speech and various testimonies of patients’ populations on social media.

It has been suggested that the measurement of HRQoL can benefit from machine-driven, quantitative analysis of patient-generated data, which expands hypothesis testing based on patient input regarding disease experience, lifestyle preferences, functioning, and more [12]. Opinions and advice shared on social media can provide insights on HRQoL directly from patients in real-life conditions [13].

Social media listening is the collection and interpretation of all patients’ social media conversations, which can help discover what really impacts patients’ lives [14]. Social media listening aggregates large amounts of unstructured patient-centered data points to identify behavioral patterns and obtain medical insights without infringing privacy policy and personal rights. Social media listening uses text mining and the natural language processing (NLP) approach as an algorithmic toolbox for identifying and managing texts of interest [15].

Against this background, the objective of this study was to develop an algorithm that is able to detect and measure the mentions of impact of diseases and treatments on 5 HRQoL dimensions in patient’s testimonies through the scope of social media listening.

## Methods

This study was conducted through several main steps: QoL definition, literature review, data extraction and manual treatment, annotation, preprocessing and feature engineering, modeling, and statistical analysis.

### Health-Related Quality of Life

The European Knowledge Society on Quality and HRQoL has compared the many definitions of HRQoL and discussed the existing confusions between health, QoL, HRQoL, and well-being [8]. The EQ-5D tool is recommended by the French public institute that regulates recommendations toward health products, their uses, and efficacy measurement (Haute Autorité de Santé [16,17]). The SF-36 is another validated generic medical survey investigating HRQoL, broadly used by practitioners for years. Three dimensions are always at the heart of the definitions or surveys: physical, psychological, and social. However, exploring HRQoL (especially on social media, with the spontaneous discussions of patients) can shed light on other views and aspects of an individual, including economic, spiritual, or even political matters. Therefore, in addition to the 3 constant dimensions (physical, psychological, and social dimension), 2 more dimensions were added to the methodology for their important role in one’s life, which can especially be impacted in the case of diseases. The dimension of generic activity is unavoidable in one’s life and can be limited in some health states, from taking a shower to professional activities; therefore, the aim of analyzing a 4th dimension is to detect mentions of impact on patients’ activity and autonomy, which are complementary to the physical dimension that focuses on body impairments. The 5th dimension is the financial one; according to the definitions developed by the European Knowledge Society on Quality and HRQoL, economic and personal finances are important contextual factors to patients [8]. Some can encounter bad or no insurances toward treatment costs or must pay for parallel cares or products that are not covered by their insurance. Patients can express specific health expenses or the necessity to have a specific budget because of their disease; therefore, the financial dimension covers this relation between health state and the impact on one’s finances as expressed by patients in their messages.

A previous work by Cotté et al [13] showed that posts from social media could be used to assess the impact of a disease or a treatment on HRQoL. This study, focused on the narratives of cancer patients treated with immunotherapies, highlighted that posts from patients could provide additional information on HRQoL to conventional QoL measurement instruments (ie, QLQ-C30 [Quality of Life Questionnaire] and FACT-G [Functional Assessment of Cancer Therapy—General]).

### Literature Review

We searched on PubMed and Google Scholar for articles responding to the following keywords: (natural language processing[MeSH Terms] OR processing natural language[MeSH Terms]) AND (quality of life[MeSH Terms] OR health related quality of life[MeSH Terms] OR healthrelated quality of life[MeSH Terms] OR HRQOL[MeSH Terms] OR cost of illness[MeSH Terms] OR disease burden[MeSH Terms] OR sickness impact profile[MeSH Terms]). The selected results were based on NLP, social media, patients’ messages, QoL, diseases, and side effects. About 40 articles were found and used with the aim of establishing the best method and modeling to adopt (Multimedia Appendix 1). A focus was made on articles that developed machine learning techniques over neural network because of their lower cost in resources and correspondence with our database. The takeaway from literature review is that some machine learning methods, tools, or approaches were highlighted for their good performance in the literature review, such as Naive Bayes, Max Entropy, Decision Tree (10 folds), and MaxVote. AdaBoost has been used for its performance boost in the learning phases. The overall performances showed that the combination of a binary classifier was better than the use of only 1 predictive model. Concerning the supervised learning for text classification, a stacked generalization method, such as SVM-L (support vector machine light), SVM-R (support vector machine regression), GBDT (gradient boosting decision tree), Unigram, bigrams, POS (part of speech), and TF-IDF (term frequency-inverse document frequency), has proven interesting for obtaining state-of-the-art results [18].

### Data Sources and Manual Treatment

The sources of data were 19 online general or health-related community forums in France, which are as follows: Atoute [19], Doctissimo [20], AuFeminin [21], Journal des femmes [22], Psychoactif [23], Forum.hardware [24], Lesimpatientes [25], Laxophobie [26], Magic maman [27], thyroide [28], forum ado/public.fr [29], Onmeda [30], Psychologies [31], MeaMedica [32], Futura-sciences [33], Allodocteurs [34], Vulgaris Medical [35], Lymphome espoir [36], and Maman pour la vie [37]. Facebook and Twitter were not included because tweets are limited to 240 characters, which limited the probability of disease history development and impact testimonies. Facebook was also discarded for data privacy questions and difficulties of access. Messages were extracted using a web crawler technology [38,39]. Health-related messages were selected based on a named entity recognition (NER) module. NER is a process where a sentence is parsed through to find entities (names, organizations, locations, and quantities). The NER module was used here to identify drug or disease mentions using an approximate matching algorithm. These messages were then preprocessed and stored. The metadata extracted along with the text were the date and hour of post.

Raw data sets were composed of randomly selected health-related messages according to the presence of treatment or disease in it. Preprocessing of the extracted data included a code attribution to every message as identifier, the detection of sentences, normalization, and deduplication; since the extracted data were unstructured, this was a necessary first step to process patients’ posts.

### Annotation

The corpus (n=1399 posts), with 1000 (71%) posts with disease mentions and 399 (29%) posts with treatment mentions, was first manually annotated. Manual annotation was performed by 2 individuals: a health-specialized data scientist and a health care professional specialized in social media listening, both sensitized and trained about the medical field of QoL, following guidelines in accordance with the methodology of HRQoL. The 2 annotators’ profiles worked in synergy in the approach of data annotation with a medical finality. Medical insight toward patients’ testimonies was brought by one of the annotators, with an expert eye toward the variables to be included in the future models by the other. The 1399 health-related messages extracted from forums were split into 2 sets for labelling; respectively, 900 and 499 messages for the 2 annotators. The aim of this step was to classify the messages according to 5 specific dimensions corresponding to 5 different types of impact: physical, psychic, activity-related, relational, and financial. The labels data were either “not impacted” or “impacted.” If “impacted,” the concerned dimensions were characterized through annotation, and the patients’ expressions of the said impact were extracted. This collection allowed the identification of specific features for each dimension being impacted, capturing the patients’ vocabulary when mentioning the impact. To evaluate the annotation homogeneity, a subset of 100 messages coming from the data scientist’s data set was blindly annotated by the health care specialist, allowing to calculate the kappa coefficient. The kappa coefficient for interrater reliability for the presence of a general HRQoL impact was 0.724; for the physical impact, it was 0.871; for the psychic impact, 0.663; for the activity-related impact, 0.639; and for the relational impact, 0.649; this is while no messages mentioned a financial impact in this subset (κ=0). Thus, agreement ranged from strong to very strong according to the kappa Cohen coefficient scale for 4 of the dimensions, but not for the financial one because no financial impact was mentioned in the subset of messages. This high interrater reliability for 4 of the 5 dimensions suggests that the used guidelines and training about the HRQoL ensured a homogeneous annotation of the messages.

### Preprocessing and Feature Engineering

All impact-related messages were used to generate dimension-specific features. Other features were based on the message structure, such as expressed sentiment (eg, positive, negative, anger, disgust, fear, joy, sadness, and surprise), grammar (eg, count of pronouns, who is writing, and negative sentences), and conjugation (eg, count of verb tenses). A lexical field score corresponding to each HRQoL dimension was computed by counting the associated expressions previously collected during the annotation stage. We used the R packages of the Detec’t extractor [39,40] to create lexical variables. This phase enabled the development of specific models of impact detection per dimension. The rationale behind this process was to be able to adapt to the many expressions of the patients. Psychic impacts and physical impacts are different, and so are the expressions used to describe them. Hence, having specific models by dimension is a way to minimize an interpretation bias.

We ended the process with a data set or corpus composed of quantitative features such as expressed sentiments (from the Linguistic Inquiry and Word Count dictionary), grammar, conjugation, and lexical fields of HRQoL-related features.

### Model Selection

We used data mining and machine learning technologies to categorize and analyze retrieved data of our final corpus according to our predefined objective. As our features do not exhibit negative values, we normalized our data by dividing all feature values by their respective maximum so that all values would be somewhere between 0 and 1, thus minimizing interclass and intraclass variances. All the missing values were replaced by the median so as not to influence intraclass variance.

We obtained a first classification algorithm to determine if there was an impact on HRQoL (corresponding to the first step of manual annotation). Subsequently, we created a classification algorithm for each dimension to assess whether the impact concerned the related dimension (second step).

We used a 5-fold sequential forward floating selector with an extreme gradient boosting algorithm to select the best features combination. We tried first to maximize the model accuracy, but we ended up with several false negative cases. We finally chose the area under the curve (AUC) as our scoring method to maximize the true positive rate because we would rather have a slightly larger number of posts containing an impact, even with false positive, than missing some of these.

We chose sequential forward floating selector over LASSO (least absolute shrinkage and selection operator) to maximize the ROC (receiver operating characteristic) value, while LASSO is trying to minimize the cost function. This allowed to obtain the best performances for all classes instead of the majority class.

We then tried several machine learning algorithms, the K-Nearest Neighbors, SVM, Multi-Layer Perceptron, Random Forest, and finally XGBoost.

Except for the psychic dimension, XGBoost was far above the other methods in terms of AUC (Table 1).

We then performed a 5-fold cross-validated grid search on our selected features to tune our hyperparameters. We split our training set into 5 samples and trained the algorithm successively on 4 of these samples, while the last sample was used as validation set. This method allowed minimizing overfitting and making sure that the models generalize well. We varied the learning rate, the number of epochs, the number of trees and their maximum depth, the minimum weight needed in a child node, the minimum loss reduction required to make a further partition on a leaf node, and the L1 regularization. LASSO regression was preferable for feature selection in case of a great number of features, making nonimportant features even more insignificant in term of weights.

**Table 1:** AUC (area under the curve) values of the different machine learning methods.

Algorithm	Impact^a^	Physical	Psychic	Activity	Relational	Financial
KNN^b^	69.9	66.5	64.9	64.4	68.6	65.6
SVM^c^	67.9	63.3	56.3	55.3	57.7	56.9
MLP^d^	74.6	67.9	61.6	64.5	64.5	58.6
RF^e^	75	70.5	70.7	71.8	70.9	69
XGB^f^	78.5	75	71	76	71.7	76.5

^a^At least 1 impact on at least 1 of the 5 dimensions.

^b^KNN: K-Nearest Neighbors.

^c^SVM: support vector machine.

^d^MLP: Multi-Layer Perceptron.

^e^RF: Random Forest.

^f^XGB: XGBoost.

This process allowed elaborating a model that can detect a general impact. The developed algorithm filtered the corpus of messages into 2 categories: HRQoL impacted or not. For each model, we selected the relevant variables by applying the sequential forward floating selector and chose which combination could better separate an impact message from a nonimpact message. In a nutshell, it removes or adds one feature at a time on the classifier and test performances until it reaches the best possible score. The same steps were then reproduced in each dimension according to their specific features in order to obtain specific algorithms fitted for each dimension.

Features of patient expressions specific to each impact were identified with the Linguistic Inquiry and Word Count dictionary, which provides expressions for various feelings, such as positivity, negativity, joy, sadness, disgust, surprise, fear, and anger. The frequency of these expressions within the posts was used to select the relevant variables for each impact domain (Table 2). Patterns identified during data labelling were also used to select relevant variables. We can assume than to describe daily actions and difficulties, the present tense is the most appropriate tense. Conversely, to talk about an impact within the family, “we” is more often used.

Due to the lack of a specific dimension’s impact mention, some classes were imbalanced regarding one another; in order to correct that, we created an artificially balanced class by using the oversampling method SMOTE (synthetic minority oversampling technique) [41]. Based on the mathematical structure of the under-represented messages, this technique artificially creates similar examples that fit the same feature pattern in order to balance the categories. We used this method for the activity-related, relational, and financial impact algorithms.

**Table 2:** Most important features by model.

Dimension	Feature 1	Feature 2	Feature 3
Impact	Number of infinitive verbs	Count of first person of singular markers	Counted sadness expressions
Physical	Counted physical related expressions	Counted negative expressions	Number of negations
Psychic	Counted psychic-related expressions	Counted anger expressions	Counted fear expressions
Activity-Related	Counted professional, academic, or daily activity-related expressions	Count of verbs in present tense	Number of pronouns
Relational	Counted relational expressions	Count of first person of plural markers	Count of past participle verbs
Financial	Count of financial expressions	Number of “not”	Count of verbs in past tense

### Statistical Analysis

We used sensitivity (defined as correctly identifying an HRQoL impact when classified as so by our algorithm) and specificity (defined as correctly identifying a message without impact when classified as so by our algorithm). The ROC curve and the AUC were considered to measure the overall performance of the algorithm. The ROC curve represented the true positive rate (sensitivity) plotted in function of the false positive rate (100-specificity) for different thresholds of the metric.

## Results

### Corpus

We extracted 20,000 messages from health-related forums mentioning diverse and different diseases such as cancers, diabetes, endometriosis, and psychological afflictions, from defined diagnosis to syndrome name (eg, nausea, “feeling blue/depressed”). Treatments such as vaccines, Levothyrox (thyroid hormones) and psychiatric drugs were also mentioned. The goal was to constitute a representative panel of health impairments, including physical, psychological, frequent, rare, light, and heavy afflictions. This corpus merged random messages mentioning 1280 medical terms (at least 1 term per message, disease, or medication). The diseases and treatment terms were identified with exact matching methods on MedDRA (Medical Dictionary for Regulatory Activities). Of the 20,000 extracted messages posted from 2000 to 2019, we randomly selected 3000 (15%) messages, which were split into 1000 and 2000. We removed duplicate entries so that we finally annotated 1399 messages: 1000 (71%) related to diseases and 399 (29%) to treatments. In the end, we had 818 (58%) messages showing at least 1 impact on QoL, 442 (31%) showing physical impact, 519 (37%) psychic, 363 (25%) activity-related, 193 (13%) relational, and 69 (4%) financial (Table 3). Many impacts on more than 1 dimension can be expressed in messages by patients.

The final corpus was then composed of 1399 French forum messages extracted from 19 conversation threads. These messages were written by users in an informal style. The length ranged from a few words to narratives longer than 1000 characters, the average message length being 905 (SD 1041) characters.

**Table 3:** Number of messages showing health-related quality of life impact, at least 1 impact, and by dimension.

Dimension	Message, n (%)
At least 1 impact	818 (58)
Physical	442 (31)
Psychic	519 (37)
Activity-Related	363 (25)
Relational	193 (13)
Financial	69 (4)

### Modeling

From our 1399 annotated messages, we chose to split them in a 70:30 ratio where 70% of the messages were used for the training phase and the rest as validation. Out of the 1399 messages, 420 (30%) were used to evaluate the model. Among these 420 messages, 203 (48%) were predicted with an impact.

We searched for lexical fields in order to evaluate the attribution of a score per dimension. We tested the different machine learning algorithms to optimize the parameters and the results. Extreme gradient boosting was the chosen model for both impact detection and specific dimension identification. The final HRQoL impact detection algorithm was composed of several models, including a model that identified the presence of an impact and all the impact-flagged messages, which went through each specific dimension model. The models were trained using cross-validation and hyperparameter optimization. Oversampling was used to augment infrequent dimensions. This allowed us to detect a general impact on HRQoL with a sensitivity of 0.8 and a specificity of 0.7 (Table 4). Overall, 818 messages presented an impact and 581 did not. For physical impact, sensitivity was 0.56, and specificity was 0.857; for psychic impact, 0.58 and 0.828; for activity-related impact, 0.71 and 0.79; for relational impact, 0.675 and 0.73; and for financial impact, 0.77 and 0.814, respectively.

**Table 4:** Overall results of the different HRQoL^a^-impacted dimensions.

Dimension	F-measure	ROC^b^ curve
At least 1 impact	78.6	78.5
Physical	70	75
Psychic	68	71
Activity-Related	75	76
Relational	70.6	71.7
Financial	76	76.5

^a^HRQoL: health-related quality of life.

^b^ROC: receiver operating characteristic.

## Discussion

### Principal Findings

We developed an algorithm to evaluate the impact diseases and treatments can have on patients’ HRQoL based on their emotions and opinions shared on social media. The algorithm was based on an adaptation for the social media listening approach, of the EQ-5D and SF-36 scales, which are recommended by several national and international institutions for assessing HRQoL and whose psychometric proprieties are well known [7,42,43]. Five dimensions of impact on HRQoL were then covered and identified in a filtered corpus of 1399 messages. The algorithm was able to detect different types of disease and treatment impact on HRQoL with good sensitivity and specificity. The algorithm had an ROC score of 0.785 for detecting at least 1 impact on at least 1 of the 5 dimensions (0.75 for physical dimension, 0.71 for psychic, 0.76 for activity-related, 0.717 for relational, and 0.765 for financial). Compared to other studies [44,45], these indicators were high and robust; for example, with Twitter and Facebook data, the area under the curve of Caster et al [44] varied between 0.43 and 0.67. For patient forum posts, sensitivity was 0.14 (and specificity was 0.88); and for Twitter and Facebook, sensitivity was 0.08 or lower. However, the objectives and approaches of these studies were different from ours, and it is thus quite difficult to compare the results. Performance might vary according to the data source. Considering that we were able to access a large data set and to use a satisfying training subset, this might explain our better performance. Nonetheless, Facebook and Twitter were discarded from our extracted sources due to the short messages of Twitter and the difficulties of access to Facebook data.

Social media listening allows direct monitoring of patients’ messages capturing “live” their opinions and feelings compared to a punctual “fixed” self-administered questionnaire. This approach corresponds more to the evolutive nature of HRQoL.

Our study adds to the literature on the use of NLP and text mining concerning medical care from web-based data. This approach relies on the potential strength of large and real time web-based data, which are complementary to classic medical reporting systems. This work contributes to the need for an improvement in methodologies that can produce more sophisticated joint models of user and message-level information or the use of syntactic structure as their features.

A similar study was conducted to outpredict baselines of popular happy and hedonistic lexica through the satisfaction with life scale over Facebook volunteers [46]. The findings of this study were also encouraging by demonstrating the effectiveness of machine learning algorithms to detect users’ health-related emotions.

Another study carried out in France [47] showed a good performance in terms of sensitivity and specificity of an NLP method to detect self-reported signals of issues with treatments. Our results confirm the same success of established statistical detection algorithms in social media for a wide range of diseases and treatments.

### Strengths and Limitations

This methodological study contributes to the growing research on social media listening and machine learning in general as a technique to develop and train tools to measure broad constructs such as HRQoL. Our work is among the first research projects proving that a social media listening tool can provide a sound and efficient measurement of impacts on HRQoL directly accessible from patients to health professionals. In this sense, it highlights some of the promises of social media and forums as data sources. One of the strengths of this study was the quality of preprocessing and processing of the data extracted. Several cleansing and validation steps were performed to ensure the quality of the messages. Furthermore, we used medically validated (general) scales, the EQ-5D and SF-36, as a strong scientific basis and gold standard for the detection of 5 specific HRQoL dimensions (ie, physical, psychological, activity-related, financial, and social). Different diseases or treatments would differently affect patients; therefore, our generalist approach of the machine learning model, which has been trained based on the patients’ free speech on various diseases and treatments, is able to detect different expressions of impact on our 5 common dimensions.

However, an algorithm does not have the human sensitivity to understand very specific and subjective ways of expressing a HRQoL impact (such as sarcasm), despite the constant improvement of the work. Sentimental analysis can complement such algorithms, and manual review remains strongly required. Additionally, our approach lacks flexibility in the feature extraction process; impact-specific features are not exhaustive because the expression of impact can vary. This also requires improvement in order to complete the lexical fields.

Limitations also include the data sources. More analysis is needed to prove that insights from social media are complementary to a patient-centric repository. Furthermore, Twitter and Facebook were discarded as sources due to short message format and accessibility issues; however, this does not mean that these social platforms are irrelevant resources for analyzing health testimonies from patients.

Our data were randomly extracted from a large sample of French messages coming from French forums and social media. The fact that our sample selection was random should ensure a certain representativity of the internet message population. The proportion of women speaking about their health in forums is higher than the proportion of men (difference of 6%) [48], which introduces a possible bias when exploring HRQoL. However, our algorithm is designed to work on data coming from French forums and social media with similar gender proportions.

Future work is needed to continue training the algorithm and to further study the differences on HRQoL between internet users and patients not posting messages on social media or forums.

### Implications

We provided evidence that social media listening can be used to assess the impact and burden of one or more diseases and treatments on patients’ HRQoL. These findings can provide public health experts, health care professionals, and pharmaceutical companies with patient-generated information on their experiences with treatments, burden of diseases, and needs for appropriate medical care in a timely manner and in real-life conditions. For instance, the generated data coming directly from patients can inform potential changes of a treatment and development of new pharmaceutical products. The use of social media listening might be recommended to monitor HRQoL constantly and consistently in patients under a new treatment or experiencing a severe disease.

### Conclusion

We developed an algorithm that can translate social media patient messages into the identification of an impact on HRQoL. Based on medically validated questionnaires, this is a patient-centered approach using machine learning and NLP to better understand how diseases and treatments can represent a burden for patients.

## Appendix

Multimedia Appendix 1 Literature references.

## Abbreviations

AUC: area under the curve
EQ-5D: Euro Quality of Life 5 Dimensions
FACT-G: Functional Assessment of Cancer Therapy—General
GBDT: gradient boosting decision tree
HRQoL: health-related quality of life
LASSO: least absolute shrinkage and selection operator
MedDRA: Medical Dictionary for Regulatory Activities
NER: named entity recognition
NLP: natural language processing
POS: part of speech
QLQ-C30: Quality of Life Questionnaire
QoL: quality of life
ROC: receiver operating characteristic
SF-36: Short Form Health Survey
SMOTE: synthetic minority oversampling technique
SVM-L: support vector machine light
SVM-R: support vector machine regression
TF-IDF: term frequency-inverse document frequency
