# Supplementary material for: A New Method to Extract Health-Related Quality of Life Data From Social Media Testimonies: Algorithm Development and Validation
Source: J Med Internet Res. 2022 Jan 28;24(1):e31528. doi: 10.2196/31528 (PMC8838601; doi:10.2196/31528)
Supplement: Multimedia Appendix 1 [file jmir_v24i1e31528_app1.docx]

Appendix 1 - Litterature

« Automated Assessment of Patients’ Self-Narratives for Posttraumatic Stress Disorder Screening Using Natural Language Processing and Text Mining - Qiwei He, Bernard P. Veldkamp, Cees A. W. Glas, Theo de Vries, 2017 ». Consulté le 21 septembre 2021. <https://journals.sagepub.com/doi/abs/10.1177/1073191115602551>.

Blake, C., et W. Pratt. « Better rules, fewer features: a semantic approach to selecting features from text ». In *Proceedings 2001 IEEE International Conference on Data Mining*, 59‑66, 2001. <https://doi.org/10.1109/ICDM.2001.989501>.

Bollegala, Danushka, Simon Maskell, Richard Sloane, Joanna Hajne, et Munir Pirmohamed. « Causality Patterns for Detecting Adverse Drug Reactions From Social Media: Text Mining Approach ». *JMIR Public Health and Surveillance* 4, n^o^ 2 (9 mai 2018): e8214. <https://doi.org/10.2196/publichealth.8214>.

Booth, Alison, Timothy Bell, Sonia Halhol, Shiyu Pan, Verna Welch, Evie Merinopoulou, Dimitra Lambrelli, et Andrew Cox. « Using Social Media to Uncover Treatment Experiences and Decisions in Patients With Acute Myeloid Leukemia or Myelodysplastic Syndrome Who Are Ineligible for Intensive Chemotherapy: Patient-Centric Qualitative Data Analysis ». *Journal of Medical Internet Research* 21, n^o^ 11 (22 novembre 2019): e14285. <https://doi.org/10.2196/14285>.

Chan, Lili, Kelly Beers, Amy A. Yau, Kinsuk Chauhan, Áine Duffy, Kumardeep Chaudhary, Neha Debnath, et al. « Natural Language Processing of Electronic Health Records Is Superior to Billing Codes to Identify Symptom Burden in Hemodialysis Patients ». *Kidney International* 97, n^o^ 2 (février 2020): 383‑92. <https://doi.org/10.1016/j.kint.2019.10.023>.

Chen, Xiaoyi, Carole Faviez, Stéphane Schuck, Agnès Lillo-Le-Louët, Nathalie Texier, Badisse Dahamna, Charles Huot, et al. « Mining Patients’ Narratives in Social Media for Pharmacovigilance: Adverse Effects and Misuse of Methylphenidate ». *Frontiers in Pharmacology* 9 (2018): 541. <https://doi.org/10.3389/fphar.2018.00541>.

Cheng, Qijin, Tim MH Li, Chi-Leung Kwok, Tingshao Zhu, et Paul SF Yip. « Assessing Suicide Risk and Emotional Distress in Chinese Social Media: A Text Mining and Machine Learning Study ». *Journal of Medical Internet Research* 19, n^o^ 7 (10 juillet 2017): e7276. <https://doi.org/10.2196/jmir.7276>.

« Choosing Your Platform for Social Media Drug Research and Improving Your Keyword Filter List - Nikki Adams, Eleanor Erin Artigiani, Eric D. Wish, 2019 ». Consulté le 21 septembre 2021. <https://journals.sagepub.com/doi/abs/10.1177/0022042619833911>.

Cocos, Anne, Alexander G. Fiks, et Aaron J. Masino. « Deep Learning for Pharmacovigilance: Recurrent Neural Network Architectures for Labeling Adverse Drug Reactions in Twitter Posts ». *Journal of the American Medical Informatics Association: JAMIA* 24, n^o^ 4 (1 juillet 2017): 813‑21. <https://doi.org/10.1093/jamia/ocw180>.

Conway, Mike, Mengke Hu, et Wendy W. Chapman. « Recent Advances in Using Natural Language Processing to Address Public Health Research Questions Using Social Media and ConsumerGenerated Data ». *Yearbook of Medical Informatics* 28, n^o^ 1 (août 2019): 208‑17. <https://doi.org/10.1055/s-0039-1677918>.

Cook, Nigel, Anmol Mullins, Raju Gautam, Sharath Medi, Clementine Prince, Nishith Tyagi, et Jyothi Kommineni. « Evaluating Patient Experiences in Dry Eye Disease Through Social Media Listening Research ». *Ophthalmology and Therapy* 8, n^o^ 3 (1 septembre 2019): 407‑20. <https://doi.org/10.1007/s40123-019-0188-4>.

Dasgupta, Nabarun, et Sidney H. Schnoll. « Signal Detection in Post-Marketing Surveillance for Controlled Substances ». *Drug and Alcohol Dependence* 105 Suppl 1 (1 décembre 2009): S33-41. <https://doi.org/10.1016/j.drugalcdep.2009.05.019>.

Dinakar, Karthik, Emily Weinstein, Henry Lieberman, et Robert Selman. « Stacked Generalization Learning to Analyze Teenage Distress ». *Proceedings of the International AAAI Conference on Web and Social Media* 8, n^o^ 1 (16 mai 2014): 81‑90.

Dreisbach, Caitlin, Theresa A. Koleck, Philip E. Bourne, et Suzanne Bakken. « A Systematic Review of Natural Language Processing and Text Mining of Symptoms from Electronic Patient-Authored Text Data ». *International Journal of Medical Informatics* 125 (1 mai 2019): 37‑46. <https://doi.org/10.1016/j.ijmedinf.2019.02.008>.

Eshleman, Ryan, et Rahul Singh. « Leveraging Graph Topology and Semantic Context for Pharmacovigilance through Twitter-Streams ». *BMC Bioinformatics* 17, n^o^ Suppl 13 (6 octobre 2016): 335. <https://doi.org/10.1186/s12859-016-1220-5>.

Eslami, Behnaz, Mehdi Habibzadeh Motlagh, Zahra Rezaei, Mohammad Eslami, et Mohammad Amin Amini. « Unsupervised Dynamic Topic Model for Extracting Adverse Drug Reaction from Health Forums ». *Applied Computer Science* Vol. 16, n^o^ no 1 (2020). <https://doi.org/10.23743/acs-2020-04>.

Gonzalez-Hernandez, G., A. Sarker, K. O’Connor, et G. Savova. « Capturing the Patient’s Perspective: A Review of Advances in Natural Language Processing of Health-Related Text ». *Yearbook of Medical Informatics* 26, n^o^ 1 (août 2017): 214‑27. <https://doi.org/10.15265/IY-2017-029>.

Husseini Orabi, Ahmed, Prasadith Buddhitha, Mahmoud Husseini Orabi, et Diana Inkpen. « Deep Learning for Depression Detection of Twitter Users ». In *Proceedings of the Fifth Workshop on Computational Linguistics and Clinical Psychology: From Keyboard to Clinic*, 88‑97. New Orleans, LA: Association for Computational Linguistics, 2018. <https://doi.org/10.18653/v1/W18-0609>.

Huynh, Trung, Yulan He, Alistair Willis, et Stefan Rüger. « Adverse Drug Reaction Classification With Deep Neural Networks », 877‑87. Osaka: COLING, 2016. <http://coling2016.anlp.jp/doc/main.pdf>.

Jones, Josette, Meeta Pradhan, Masoud Hosseini, Anand Kulanthaivel, et Mahmood Hosseini. « Novel Approach to Cluster Patient-Generated Data Into Actionable Topics: Case Study of a Web-Based Breast Cancer Forum ». *JMIR Medical Informatics* 6, n^o^ 4 (29 novembre 2018): e45. <https://doi.org/10.2196/medinform.9162>.

Kalyan, Katikapalli Subramanyam, et S. Sangeetha. « SECNLP: A Survey of Embeddings in Clinical Natural Language Processing ». *Journal of Biomedical Informatics* 101 (janvier 2020): 103323. <https://doi.org/10.1016/j.jbi.2019.103323>.

Karmen, Christian, Robert C. Hsiung, et Thomas Wetter. « Screening Internet Forum Participants for Depression Symptoms by Assembling and Enhancing Multiple NLP Methods ». *Computer Methods and Programs in Biomedicine* 120, n^o^ 1 (juin 2015): 27‑36. <https://doi.org/10.1016/j.cmpb.2015.03.008>.

Kim, Joo-Chang, et Kyungyong Chung. « Associative Feature Information Extraction Using Text Mining from Health Big Data ». *Wireless Personal Communications* 105, n^o^ 2 (1 mars 2019): 691‑707. <https://doi.org/10.1007/s11277-018-5722-5>.

Liu, Zengjian, Ming Yang, Xiaolong Wang, Qingcai Chen, Buzhou Tang, Zhe Wang, et Hua Xu. « Entity Recognition from Clinical Texts via Recurrent Neural Network ». *BMC Medical Informatics and Decision Making* 17, n^o^ Suppl 2 (5 juillet 2017): 67. <https://doi.org/10.1186/s12911-017-0468-7>.

Morlane-Hondère, François, Cyril Grouin, et Pierre Zweigenbaum. « Identification of Drug-Related Medical Conditions in Social Media ». In *Proceedings of the Tenth International Conference on Language Resources and Evaluation (LREC’16)*, 2022‑28. Portorož, Slovenia: European Language Resources Association (ELRA), 2016. <https://aclanthology.org/L16-1320>.

Nie, L., Mohammad Akbari, T. Li, et T.-S Chua. « A joint local-global approach for medical terminology assignment ». *CEUR Workshop Proceedings* 1276 (1 janvier 2014): 24‑27.

O’Dea, Bridianne, Stephen Wan, Philip J. Batterham, Alison L. Calear, Cecile Paris, et Helen Christensen. « Detecting Suicidality on Twitter ». *Internet Interventions* 2, n^o^ 2 (1 mai 2015): 183‑88. <https://doi.org/10.1016/j.invent.2015.03.005>.

Opitz, Thomas, Jérome Aze, Sandra Bringay, Cyrille Joutard, Christian Lavergne, et Caroline Mollevi. « Breast Cancer and Quality of Life: Medical Information Extraction from Health Forums ». *Studies in Health Technology and Informatics* 205 (2014): 1070‑74.

Park, Jungsik, et Young Uk Ryu. « Online Discourse on Fibromyalgia: Text-Mining to Identify Clinical Distinction and Patient Concerns ». *Medical Science Monitor: International Medical Journal of Experimental and Clinical Research* 20 (7 octobre 2014): 1858‑64. <https://doi.org/10.12659/MSM.890793>.

Park, So Hyun, et Song Hee Hong. « Identification of Primary Medication Concerns Regarding Thyroid Hormone Replacement Therapy From Online Patient Medication Reviews: Text Mining of Social Network Data ». *Journal of Medical Internet Research* 20, n^o^ 10 (24 octobre 2018): e11085. <https://doi.org/10.2196/11085>.

Paul, Michael J., Abeed Sarker, John S. Brownstein, Azadeh Nikfarjam, Matthew Scotch, Karen L. Smith, et Graciela Gonzalez. « Social media mining for public health monitoring and surveillance ». In *Biocomputing 2016*, 468‑79. WORLD SCIENTIFIC, 2015. <https://doi.org/10.1142/9789814749411_0043>.

Portier, Kenneth, Greta E. Greer, Lior Rokach, Nir Ofek, Yafei Wang, Prakhar Biyani, Mo Yu, et al. « Understanding Topics and Sentiment in an Online Cancer Survivor Community ». *Journal of the National Cancer Institute. Monographs* 2013, n^o^ 47 (décembre 2013): 195‑98. <https://doi.org/10.1093/jncimonographs/lgt025>.

Powell, Gregory E., Harry A. Seifert, Tjark Reblin, Phil J. Burstein, James Blowers, J. Alan Menius, Jeffery L. Painter, et al. « Social Media Listening for Routine Post-Marketing Safety Surveillance ». *Drug Safety* 39, n^o^ 5 (mai 2016): 443‑54. <https://doi.org/10.1007/s40264-015-0385-6>.

Ransohoff, Julia D., Azadeh Nikfarjam, Erik Jones, Brian Loew, Bernice Y. Kwong, Kavita Y. Sarin, et Nigam H. Shah. « Detecting Chemotherapeutic Skin Adverse Reactions in Social Health Networks Using Deep Learning ». *JAMA Oncology* 4, n^o^ 4 (1 avril 2018): 581‑83. <https://doi.org/10.1001/jamaoncol.2017.5688>.

Ravoire, Sophie, Marie Lang, Elena Perrin, Antoine Audry, Pascal Bilbault, Michael Chekroun, Lauren Demerville, et al. « Intérêts et limites des communautés virtuelles de patients pour la recherche sur les produits de santé ». *Therapies*, XXXIIes Rencontres Nationales de Pharmacologie et de Recherche Clinique, pour l’Innovation Thérapeutique et l’Evaluation des Technologies de Santé - Tables rondes Giens, 2 au 3 octobre 2016, 72, n^o^ 1 (1 février 2017): 125‑34. <https://doi.org/10.1016/j.therap.2016.11.055>.

Sarma, Karthik V., Brennan M. R. Spiegel, Mark W. Reid, Shawn Chen, Raina M. Merchant, Emily Seltzer, et Corey W. Arnold. « Estimating the Health-Related Quality of Life of Twitter Users Using Semantic Processing ». *Studies in Health Technology and Informatics* 264 (21 août 2019): 1065‑69. <https://doi.org/10.3233/SHTI190388>.

Schwartz, H. Andrew, Maarten Sap, Margaret L. Kern, Johannes C. Eichstaedt, Adam Kapelner, Megha Agrawal, Eduardo Blanco, et al. « PREDICTING INDIVIDUAL WELL-BEING THROUGH THE LANGUAGE OF SOCIAL MEDIA ». *Pacific Symposium on Biocomputing. Pacific Symposium on Biocomputing* 21 (2016): 516‑27.

Wang, Yanshan, Sijia Liu, Naveed Afzal, Majid Rastegar-Mojarad, Liwei Wang, Feichen Shen, Paul Kingsbury, et Hongfang Liu. « A Comparison of Word Embeddings for the Biomedical Natural Language Processing ». *Journal of Biomedical Informatics* 87 (1 novembre 2018): 12‑20. <https://doi.org/10.1016/j.jbi.2018.09.008>.

Wei, Qiang, Zongcheng Ji, Zhiheng Li, Jingcheng Du, Jingqi Wang, Jun Xu, Yang Xiang, et al. « A study of deep learning approaches for medication and adverse drug event extraction from clinical text ». *Journal of the American Medical Informatics Association* 27, n^o^ 1 (1 janvier 2020): 13‑21. <https://doi.org/10.1093/jamia/ocz063>.

Wu, Stephen, Kirk Roberts, Surabhi Datta, Jingcheng Du, Zongcheng Ji, Yuqi Si, Sarvesh Soni, et al. « Deep Learning in Clinical Natural Language Processing: A Methodical Review ». *Journal of the American Medical Informatics Association: JAMIA* 27, n^o^ 3 (1 mars 2020): 457‑70. <https://doi.org/10.1093/jamia/ocz200>.

Yang, Fu-Chen, Anthony J. T. Lee, et Sz-Chen Kuo. « Mining Health Social Media with Sentiment Analysis ». *Journal of Medical Systems* 40, n^o^ 11 (novembre 2016): 236. <https://doi.org/10.1007/s10916-016-0604-4>.

Young, Ian James Bruce, Saturnino Luz, et Nazir Lone. « A Systematic Review of Natural Language Processing for Classification Tasks in the Field of Incident Reporting and Adverse Event Analysis ». *International Journal of Medical Informatics* 132 (1 décembre 2019): 103971. <https://doi.org/10.1016/j.ijmedinf.2019.103971>.

Zhang, Min, et Guohua Geng. « Adverse Drug Event Detection Using a Weakly Supervised Convolutional Neural Network and Recurrent Neural Network Model ». *Information* 10, n^o^ 9 (septembre 2019): 276. <https://doi.org/10.3390/info10090276>.
